# The second-order coherence analysis of number state propagation through dispersive non-Hermitian multilayered structures

**DOI:** 10.1038/s41598-024-55777-0

**Published:** 2024-03-05

**Authors:** Elnaz Pilehvar, Ehsan Amooghorban, Mohammad Kazem Moravvej-Farshi

**Affiliations:** 1 Nano Plasmo-Photonic Research Group, Faculty of Electrical and Computer Engineering, Tarbiat Modares University, P.O. Box 14115-194, Tehran, 1411713116 Iran; 2 Faculty of Science, Department of Physics, Shahrekord University, P.O. Box 115, Shahrekord, 88186-34141 Iran; 3 Nanotechnology Research Group, Shahrekord University, Shahrekord, Iran

**Keywords:** Quantum optics, Single photons and quantum effects

## Abstract

To examine the second-order coherence of light propagation of quantum states in arbitrary directions through dispersive non-Hermitian optical media, we considered two sets of non-Hermitian periodic structures that consist of gain/loss unit cells. We show that each batch can satisfy the parity-time symmetry conditions at a distinct frequency. We then varied the gain/loss strength in the stable electromagnetic regime to evaluate the transmittance of N-photon number states through each structure. The results show both sets preserve their antibunching characteristics under specific incident light conditions. Furthermore, s(p)-polarized light exhibits higher (lower) second-order coherence at larger incident angles. In addition, the antibunching features of the transmitted states degrade with an increase in the number of unit cells in multilayered structures for both polarizations.

## Introduction

Under particular conditions—i.e., parity-time (PT) symmetric conditions—a non-Hermitian Hamiltonian can retain an entirely real spectrum of eigenvalues that is usually believed exclusively to belong to Hermitian systems^1–3^. PT symmetry can influence systems' functions like nonlinearity^4–6^, lasing^6,7^, switching^8^, unidirectional invisibility^9^, and non-trivial topologies^10,11^. Such systems employ a photonic platform and set the stage for the development of other research fields, such as atomic^12–14^ and plasmonic systems^15^, and electronic circuits^16,17^. These studies concentrated on classical electromagnetic waves. However, quantized light fields in PT-symmetric optical systems have received little attention.

The incident light of a nonclassical nature has some specific features, such as reduced noise and strong correlations, which can only be described in the framework of the second quantization of the electromagnetic fields. Quantum lights have found numerous applications, ranging from quantum communications and quantum computing to enhanced sensing^18^. Up to now, only a few research groups have studied PT-symmetric optical systems beyond the first quantization step^12,16,19–26^. In our most recent work^26^, we extensively studied the obliquely incident s- and p-polarized squeezed coherent state of light after transmitting through dispersive non-Hermitian multilayered structures, particularly at discrete frequencies, where the medium holds PT symmetry. Thus, exploring the quantumness of the light states can be a suitable probe for implementing PT symmetry in quantum optics. Our findings, based on first-order quantum correlations, show that PT symmetry cannot be implemented at any arbitrary angle of incidence for either polarization in quantum optics, as far as the squeezing feature of the outgoing light is concerned. This situation can change if one only probes the sub-Poissonian photon statistics of outgoing light from a non-Hermitian structure whose gain layer emission frequency is far from the incident frequency, which is a non-resonant structure. Therefore, the possibility of implementing PT symmetry with quantized fields depends on the desired nonclassical features and the resonant (non-resonant) type of structures realizing the symmetry

Motivated by these recent studies and the prominent role of higher-order coherence in quantum optics research, we focus on the second-order coherence of *N*-photon number states of s(p)-polarized lights obliquely propagating across two resonant and non-resonant sets of non-Hermitian multilayered structures, which enables PT symmetry at two specific frequencies. These allow us to investigate the concept of higher-order coherence and the role of the nature of quantized light on PT symmetry. The second-order coherence reflects the bunching (antibunching) characteristic of photons exiting the structures, which can be a strong signature of classical (nonclassical) light. Active materials can manifest electromagnetic instabilities, that is, the branch points in the complex ω-plane, where the field amplitudes grow exponentially in the time domain. When a non-Hermitian structure is electromagnetically stable, we study the influence of dispersion, loss(gain)-induced noise, and polarization of light on the antibunching feature of the transmitted light while varying the loss (gain) strength and angle of incidence. Examination of the antibunching behaviour of the transmitted light can help us more accurately assess PT symmetry in the quantum optics realm.

## Method

### Multilayered structure

**Figure. 1:**
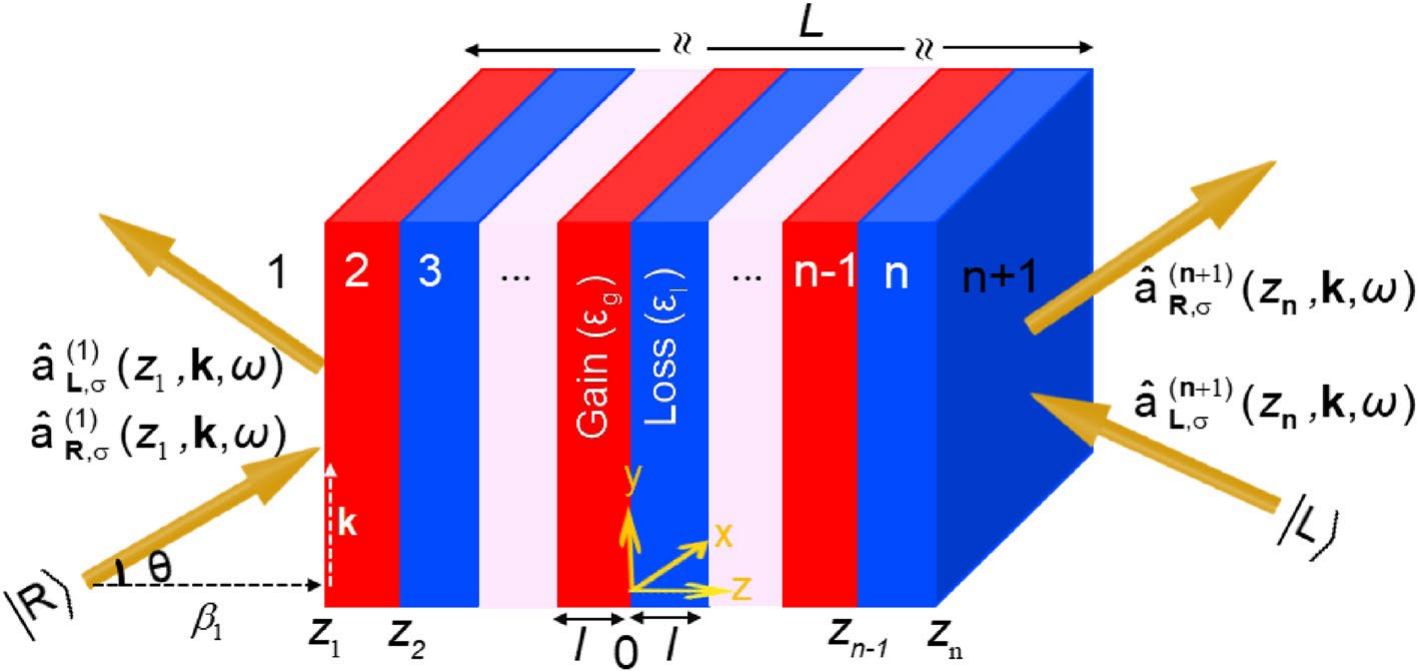
A 3D representation of a multilayered non-Hermitian structure consists of alternating gain and loss slabs with permittivities ε_g_ and ε_l_, respectively, and the same thicknesses of *l* along the z-direction. The arrows with the annihilation operators show the input and output modes.

Consider an infinitely broad dispersive non-Hermitian multilayered structure composed of {(*n* − 1)/2} pairs of homogeneous gain/loss nanolayers of the same thickness, *l* (Fig. 1). In other words, the total thickness of the multilayer embedded in a vacuum along the *z*-direction is *L* = (*n* − 1)*l*. The oblique arrows labeled with bosonic operators indicate the input and output modes.

We use a similar relation as in^27,28^ to define the dispersive relative dielectric function of the gain (*ε*_*g*_) and loss (*ε*_l_) media following the Lorentzian formula:

1 $$\varepsilon_{{\user2{g(l)}}} \left( \omega \right) = \varepsilon_{{\user2{bg(l)}}} - \frac{{\alpha_{{\user2{g(l)}}} \,\omega_{{\user2{0g(l)}}} \;\gamma_{{\user2{g(l)}}} }}{{\omega^{2} - \omega_{{0\user2{g(l)}}}^{2} + i\omega \,\gamma_{{\user2{g(l)}}} }},$$

where label *b* indicates the background materials’ relative dielectric constant; *ω,* *ω*_0g(l)_, *α*_g(l)_, and *γ*_g(l)_ denote the frequency of the input light beam, material resonance frequency, the gain (loss) coefficient, and emission (absorption) linewidth of the gain (loss) nanolayers, respectively.

Based on the principle of causality, we consider *α*_g_ < 0 (*α*_l_ > 0) and *γ*_g_ > 0 (*γ*_l_ > 0). The dielectric functions of the nanolayers should satisfy *ε* (*z,* ω) = *ε*^∗^ (−*z,* ω) to guarantee the necessary condition for the establishment of PT symmetry. Notice that the PT symmetry condition for a dispersive optical medium can be satisfied only for a discrete set of real frequencies^29^. One may practically realize such structures by employing plasmonic metamaterials, as suggested by^30–32^ as an example, grown on a lossless glass substrate with insignificant quantum noise flux.

### The exact multilayer theory

Consider a quantum state of light with s(p)-polarization obliquely striking the multilayer with the incident angle *θ* from the left (right). Rendering the second quantization formalism of electromagnetic fields, we write the transverse component of the electric field operator in the jth layer for *ω* > 0^33–36^:

2 $$\begin{gathered} {\varvec{E}}_{\sigma }^{ + (j)} \left( {z,{\varvec{k}},t} \right) = i\int_{i\eta + 0}^{i\eta + \infty } {d\omega \,\sqrt {{{\hbar \omega } \mathord{\left/ {\vphantom {{\hbar \omega } {4\pi \varepsilon_{0} cA}}} \right. \kern-0pt} {4\pi \varepsilon_{0} cA}}} } \\ \times \left\{ {\exp \left( {i\beta^{\prime}_{j} z} \right)\hat{a}_{R,\sigma }^{(j)} \left( {z,{\mathbf{k}},\omega } \right){\mathbf{e}}_{R,\sigma }^{(j)} ({\mathbf{k}})} \right. \\ + \left. {\exp \left( { - i\beta^{\prime}_{j} z} \right)\hat{a}_{L,\sigma }^{(j)} \left( {z,{\mathbf{k}},\omega } \right){\mathbf{e}}_{L,\sigma }^{(j)} ({\mathbf{k}})} \right\}e^{ - i\omega t} . \\ \end{gathered}$$

where *β′*_*j*_ denotes the real part of the longitudinal component of the propagation constant in the *j*th layer

3 $$\beta_{j} ({\varvec{k}},\omega ) = \beta^{\prime}_{j} + i\beta^{\prime\prime}_{j} = \sqrt {\varepsilon_{j} (\omega )\omega^{2} /c^{2} - {\varvec{k}}^{2} } = \frac{\omega }{c}\sqrt {\varepsilon_{j} (\omega ) - \sin^{2} \theta } ,$$

and *ℏ,* *ε*_0,_ *c,* *A*, **k**, and ${\mathbf{e}}_{R(L),\sigma }^{(j)}$, in Eq. (2), represent the reduced Planck’s constant, the vacuum permittivity, free space light velocity, the cross-sectional area of the quantization, the in-plane wavevector in any layer, and the polarization unit vector for the right (R) and left (L) going waves with *σ* = *s*, *p*. The component ${\varvec{E}}_{\sigma }^{ - (j)} \left( {z,t} \right)$ that corresponds to the negative frequency (*ω* < 0) is the Hermitian adjoint of Eq. (2).

As we will discuss in the following section, we set *η* = max {0, Im (nonanalytic points of *β*_j_), Im (poles of transmission and right(left) reflections)} to avoid exponential growth of the field amplitude in the time domain due to instabilities^37^. We can explicitly assess the scattering of quantum light fields from the structure using the quantum input–output relations, which link the output bosonic annihilation operators $\hat{a}_{L,\sigma }^{(1)} \left( {z_{1} ,{\varvec{k}},\omega } \right)$ $\hat{a}_{R,\sigma }^{(n + 1)} \left( {z_{n} ,{\varvec{k}},\omega } \right)$ with their input operators $\hat{a}_{R,\sigma }^{(1)} \left( {z_{1} ,{\varvec{k}},\omega } \right)$ and $\hat{a}_{L,\sigma }^{(n + 1)} \left( {z_{n} ,{\varvec{k}},\omega } \right)$, and the noise operators, $\hat{F}_{R,\sigma } \left( \omega \right){\text{ and }}\hat{F}_{L,\sigma } \left( \omega \right)$, through

4a $$\left( {\begin{array}{*{20}c} {\hat{a}_{L,\sigma }^{(1)} \left( {z_{1} ,{\varvec{k}},\omega } \right)} \\ {\hat{a}_{R,\sigma }^{(n + 1)} \left( {z_{n} ,{\varvec{k}},\omega } \right)} \\ \end{array} } \right) = {\mathbb{S}}_{\sigma } \left( {\begin{array}{*{20}c} {\hat{a}_{R,\sigma }^{(1)} \left( {z_{1} ,{\varvec{k}},\omega } \right)} \\ {\hat{a}_{L,\sigma }^{(n + 1)} \left( {z_{n} ,{\varvec{k}},\omega } \right)} \\ \end{array} } \right) + \left( {\begin{array}{*{20}c} {\hat{F}_{L,\sigma } \left( {{\varvec{k}},\omega } \right)} \\ {\hat{F}_{R,\sigma } \left( {{\varvec{k}},\omega } \right)} \\ \end{array} } \right)\,,\,$$

where the elements of the scattering matrix,

4b $$\,{\mathbb{S}}_{\sigma } \equiv \left( {\begin{array}{*{20}c} {r_{\sigma ,L} } & {t_{\sigma } } \\ {t_{\sigma } } & {r_{\sigma R} } \\ \end{array} } \right),$$

analogous to classical optics are the transmission coefficients through the right and left boundaries (i*.e.,* *t*_σR_(*z* =  − *L*/2) = *t*_σL_(*z* =  + *L*/2) = *t*_σ_), and the reflection coefficients, *r*_σR_ and *r*_σL_ from the right and left boundaries of the multilayer. The quantum noise operator at the left (right) boundary, $\hat{F}_{L(R)}$, which originates from the loss (gain) layer, on the contrary, has no classical equivalent. One can find the explicit forms of *t*_σ_, *r*_σR(L)_, and the quantum noise operators in^35^. The bosonic creators and annihilators at the input and output ports satisfy the canonical commutations,

5a $$\begin{gathered} \left[ {\hat{a}_{R,\sigma }^{(1)} \left( {z,{\varvec{k}},\omega } \right),\hat{a}_{{R,\sigma^{\prime}}}^{(1)\dag } \left( {z^{\prime},\user2{k^{\prime}},\omega^{\prime}} \right)} \right] = \left[ {\hat{a}_{L,\sigma }^{(n + 1)} \left( {z,{\varvec{k}},\omega } \right),\hat{a}_{{L,\sigma^{\prime}}}^{(n + 1)\dag } \left( {z^{\prime},\user2{k^{\prime}},\omega^{\prime}} \right)} \right] \\ = \delta_{{\sigma \sigma^{\prime}}} \delta \left( {\omega - \omega^{\prime}} \right)\delta \left( {{\varvec{k}} - \user2{k^{\prime}}} \right), \\ \end{gathered}$$

5b $$\left[ {\hat{a}_{R,\sigma }^{(1)} \left( {z,{\varvec{k}},\omega } \right),\hat{a}_{{L,\sigma^{\prime}}}^{(n + 1)\dag } \left( {z^{\prime},\user2{k^{\prime}},\omega^{\prime}} \right)} \right] = 0.$$

By substituting Eq. (5) in Eq. (4), we can derive the commutation relations for the output amplitude operators, similar to Eq. (5).

## Results and discussion

Here, we consider two types of multilayers. The background materials for the loss and gain layers making the first-type unit cell (UC1) are identical. In other words, UC1 is of resonant type with Δ*ε*_b_ = (*ε*_bl_ − *ε*_bg_) = 0 and Δ*ω*_0_ = (*ω*_0l_ − *ω*_0g_) = 0. The second type of unit cell (UC2) is a nonresonant type with Δ*ε*_b_ = 1.22 and Δ*ω*_0_ = 200 Trad∙s^−1^. Table 1 lists the physical constants related to the background materials constituting UC1 and UC2 for the multilayers considered in this study (Fig. 1) that are similar to those used in^25–28^. The layer thicknesses of both unit cells are assumed to be identical (*l* = 10 nm). Moreover, the substitution of the data in Table 1 into (1) reveals that the frequency at which UC1 fulfills the necessary PT symmetry condition for any given loss value of *α*_l_ = | *α*_g_ | is *ω* = *ω*_PT1_ = *ω*_0g_. Whereas, in UC2, the necessary condition for PT symmetry is solely fulfilled for *α*_l_ = 2 and *α*_g_ = −20.86 at *ω* = *ω*_PT2_= 1.58 *ω*_0g_. For those readers with an interest in obtaining a more detailed exposition of the PT-symmetry condition, we would like to direct their attention to Appendix A of^25^.

**Table 1:** The physical constants related to the background materials constituting UC1 and UC2 for the multilayers that are considered in this work^25–28^.

Symbol	Definition	size		unit
		UC1	UC2	
ε_bl_	The lossy material background relative permittivity	2	3.22	−
ε_bg_	The active material background relative permittivity	2	2	−
γ_l_	The lossy material absorption linewidth	670	140	Trad∙s^−1^
γ_g_	The active material emission linewidth	670	670	Trad∙s^−1^
ω_0l_	The lossy material resonance frequency	1000	1200	Trad∙s^−1^
ω_0g_	The active material emission frequency	1000	1000	Trad∙s^−1^

Having all these, we consider a quantum state with *s*(*p*)-polarization impinging obliquely upon the multilayered structures at *z* = *z*_1_ (*z*_n_) from the left (right) and study its behaviour after transmitting out from *z* = *z*_n_ (*z*_1_).

### Stable and unstable regimes

The propagation of electromagnetic waves in active materials requires careful treatment because of certain instabilities that may lead to the exponential growth of electromagnetic energy in such media. In other words, an electric field may diverge at a particular position inside the gain layer due to the branch points in the refractive index and the longitudinal components of the propagation constant (*β*_*g*_) in the upper half of the complex *ω*-plane. This issue is particularly crucial when measuring the second-order coherence of the outgoing electric fields from such structures in the time domain. Moreover, there are possibilities that the transmission and reflection coefficients pose some poles, making the structure electromagnetically unstable. Nevertheless, this issue does not concern us in this study because of the parameters listed in Table 1. There are three types of instabilities: global, convective, and absolute (see^37^ for more details). Some of these instabilities can be eliminated, by limiting the transverse extent of the gain layer, considering nonlinear effects such as gain saturation in a realistic model characterizing the gain medium and the presence of the loss layer as a surrounding medium, which indeed exists in our model. In case of instabilities associated with branch points, the longitudinal component of the propagation constant of *β*_*g*_ and, in turn, other optical parameters like transmission and reflection coefficients have no physical meaning at real frequencies (i.e., when the electromagnetic field diverges in the gain medium) unless their behaviour along the branch cut is considered as well. In this manner, the electromagnetic fields at last blow up with time. Contrary to the gain medium, the longitudinal component of the propagation constant in a passive medium (*β*_l_) is always analytic in the upper half of the *ω*-plane^37–39^.

**Figure 2:**
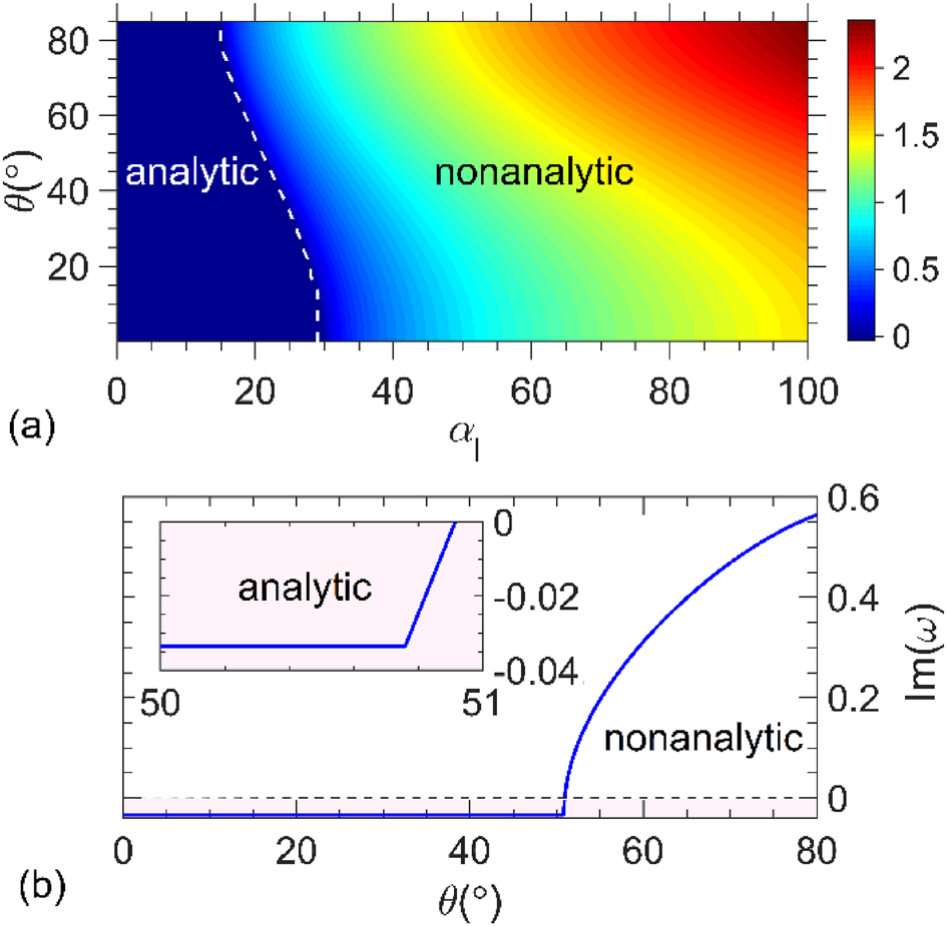
**(a)** Profile of Im *ω* extracted from zeros of expression *ε*_*j*_ (ω) − sin^2^ (*θ*) in Eq. (3) in the *α*_l_ − *θ* plane for a structure composed of a unit-cell designated by UC1. **(b)** Im ω versus θ for UC2 at *α*_l_ = 2. The inset in (**b**) shows a zoomed-in portion of the plot for the values of *θ* where β_g_ remains analytic in the complex ω-plane, that is the pink region. The dashes in both parts represent the boundaries between analytic and non-analytic regions.

As elucidated earlier, the unstable electromagnetic behavior generally arises due to the presence of gain media. In view of the PT-symmetry, which entails a spatial distribution of gain and loss in physical systems, it is plausible that the aforementioned instabilities are exhibited in PT-symmetric structures. As a consequence, utmost caution must be exercised to ensure that the system operates in a stable regime when the PT-symmetry criterion is satisfied. To avoid instabilities, we should determine the input parameters for which the solution of Eq. (3) is analytic for both unit cells. In other words, *β*_*g*_ has no branch point in the upper half of the complex *ω*-plane. Hence, we use Eq. (1) for *ε*_*j*_(ω) and solve Eq. (3) to find the branch points of *β*_g_ in the complex *ω*-plane. Figure 2a illustrates the imaginary part of *ω* (Im *ω*) as a function of the loss coefficient, *α*_l_ (= | *α*_g_ |), and incident angle, *θ*, for both *s* and *p* polarizations for UC1. Notice, that Im *ω* > 0 (< 0) represents the upper (lower) half of the complex *ω*-plane. The white dashes in Fig. 2a, representing the loci of Im *ω* = 0, indicate the boundary between the analytic and nonanalytic regions. Figure 2b illustrates Im *ω* versus *θ* for *α*_l_ = 2 and *α*_g_ = −20.86 for UC2. As can be seen in this figure, Im *ω* = 0 at *θ* = 51°, below which *β*_*g*_ is an analytical function in the complex *ω-*plane up to a particular value of *θ*.

In what follows, to exclude the above instabilities for both sets, we restrict ourselves to the gain strengths and angles of incidence in which optical parameters such as *β*_*g*_ are analytic in the upper half-plane. Thus, the structure under study was in a stable electromagnetic regime.

### The second-order coherence

In this section, we investigate how transmission through the proposed structure affects the antibunching property of the incident state. To do so, we measured the second-order correlation function of the output light in the time domain. Employing a coincidence photocount detector placed at *z* ( > *L* */* 2 ) on the right-hand side of the structure, the second-order coherence is given by ^39^:

6 $$g_{\sigma }^{(2)} \left( {z,{\varvec{k}},t,\tau } \right) = \frac{{\left\langle {{\varvec{E}}_{\sigma }^{ - (n + 1)} \left( {z,{\varvec{k}},t} \right){\varvec{E}}_{\sigma }^{ - (n + 1)} \left( {z,{\varvec{k}},t + \tau } \right){\varvec{E}}_{\sigma }^{ + (n + 1)} \left( {z,{\varvec{k}},t + \tau } \right){\varvec{E}}_{\sigma }^{ + (n + 1)} \left( {z,{\varvec{k}},t} \right)} \right\rangle }}{{\left\langle {{\varvec{E}}_{\sigma }^{ - (n + 1)} \left( {z,{\varvec{k}},t} \right){\varvec{E}}_{\sigma }^{ + (n + 1)} \left( {z,{\varvec{k}},t} \right)} \right\rangle \left\langle {{\varvec{E}}_{\sigma }^{ - (n + 1)} \left( {z,{\varvec{k}},t + \tau } \right){\varvec{E}}_{\sigma }^{ + (n + 1)} \left( {z,{\varvec{k}},t + \tau } \right)} \right\rangle }}.$$

The intensity correlations for the transmitted light, in the range of (*g*_*σ*_^(2)^(*τ* = 0) > *g*_*σ*_^(2)^(*τ*) or *g*_*σ*_^(2)^(*τ* = 0) > 1), (*g*_*σ*_^(2)^(*τ* = 0) = *g*_*σ*_^(2)^(*τ*) or *g*_*σ*_^(2)^(*τ* = 0) = 1)), and (*g*_*σ*_^(2)^(*τ* = 0) < *g*_*σ*_^(2)^(*τ*) or *g*_*σ*_^(2)^(*τ* = 0) < 1), corresponds to the so-called bunching, coherent, and antibunching of photons, respectively. We here take into account the incident state of light | *R* ⟩| *L* ⟩ where the right-going *s*(*p*)-polarized quantum state | *R* ⟩ impinging upon the structure (at *z* =  *− L* */* 2) and the traveling leftward state | *L* ⟩ (at *z* =  + *L* */* 2) are, respectively, the continuum *N*-photon-number state $\left| {N,{\varvec{k}},\xi_{\sigma } } \right\rangle$ and the conventional vacuum state | 0 ⟩. In Supplementary, we will treat another incident state of lights when the restructure is illuminated by the interchanged states | *L* ⟩| *R* ⟩, or the entangled state^40^. There, it is seen why the incident state | *R* ⟩| *L* ⟩ is an appropriate choice for examining the antibunching property of the transmitted states.

One can generate an *N*-photon number state by applying the creation operator $\hat{a}_{R,\sigma }^{(1)\dag }$ on the quantum vacuum state of the form ^33^:

7 $$\left| R \right\rangle \equiv \left| {N,{\varvec{k}},\xi_{\sigma } } \right\rangle = \frac{1}{{\sqrt {N!} }}\left[ {\int_{0}^{\infty } {d\omega \,\xi_{\sigma }^{*} \left( \omega \right)\,\hat{a}_{R,\sigma }^{(1)\dag } \left( {{\varvec{k}},\omega } \right)} } \right]^{N} \left| 0 \right\rangle ,$$

where,

8 $$\xi_{\sigma } \left( \omega \right) = \left( {{{L^{2} } \mathord{\left/ {\vphantom {{L^{2} } {2\pi c^{2} }}} \right. \kern-0pt} {2\pi c^{2} }}} \right)^{1/4} \exp \left[ {{{ - L^{2} \left( {\omega - \omega_{c} } \right)^{2} } \mathord{\left/ {\vphantom {{ - L^{2} \left( {\omega - \omega_{c} } \right)^{2} } {4c^{2} }}} \right. \kern-0pt} {4c^{2} }}} \right]$$

describes the frequency distribution of the photon number wave packet, whose typical form of an optical pulse is a Gaussian wave packet with a centre frequency *ω*_c_ and mean-square spatial length *L*^2^.

After some lengthy mathematical manipulations, we can obtain a compact formula for the second-order correlation function (Eq. 6) in region *n* + 1 for the incident state of light $\left| R \right\rangle \left| L \right\rangle = \left| {N,{\varvec{k}},\xi_{\sigma } } \right\rangle$| 0 ⟩ as

9 $$\begin{aligned} g_{\sigma }^{(2)} \left( {t_{r} ,\tau ,\theta } \right) & = \left\{ {N\left( {N - 1} \right)\left| {J_{1\sigma } \left( {t_{r} ,\theta } \right)} \right|^{2} \left| {J_{1\sigma } \left( {t_{r} + \tau ,\theta } \right)} \right|^{2} } \right. + \left| {J_{2\sigma } \left( {\tau ,\theta } \right)} \right|^{2} + J_{2\sigma }^{2} \left( {0,\theta } \right) \\ & \quad  + NJ_{2\sigma } \left( {0,\theta } \right)\left[ {\left| {J_{1\sigma } \left( {t_{r} ,\theta } \right)} \right|^{2} + \left| {J_{1\sigma } \left( {t_{r} + \tau ,\theta } \right)} \right|^{2} } \right] \\ & \quad + \left. {2N{\text{Re}} \left[ {J_{1\sigma } \left( {t_{r} ,\theta } \right)J_{1\sigma }^{*} \left( {t_{r} + \tau ,\theta } \right)J_{2\sigma } \left( {\tau ,\theta } \right)} \right]} \right\} \\ & \quad \times \left\{ {\left[ {N\left| {J_{1\sigma } \left( {t_{r} ,\theta } \right)} \right|^{2} + J_{2\sigma } \left( {0,\theta } \right)} \right]\left[ {N\left| {J_{1\sigma } \left( {t_{r} + \tau ,\theta } \right)} \right|^{2} + J_{2\sigma } \left( {0,\theta } \right)} \right]} \right\}^{ - 1} . \\ \end{aligned}$$

where *t*_r_ = *t* − *z* / *c*. Here, the dependence of g_*σ*_^(2)^ in Eq. (9) on transmitted light as a wave packet contribution is provided by *J*_1σ,_ and that of the quantum noise flux as a noise contribution is given by *J*_2σ._ We present the explicit forms of *J*_1σ_ and *J*_2σ_ in Supplementary S.1. In our calculations, we considered a Gaussian wave packet with a centre frequency *ω*_c_ = *ω*_PT_ = *ω*_0g_ (1.58*ω*_0g_) for UC1 (UC2) and an incident pulse length of *M* = 20 *l*. Moreover, we measured the delay time in terms of the mean coherence time *τ*_c_ = *M*/ *c* and maintained the structure at 0 K.

In a recent study ^25^, we extensively investigated the significance of a few specific loss/gain coefficients of a bilayer (i.e., a unit cell) at normal incidence. In that study, we showed that the anisotropic transmission resonance (ATR), at which the reflectance vanishes only for waves incident from one side of the bilayer, occurs at | *α*_g_ | = *α*_l_ = 24 for the UC1 bilayer. The unidirectional invisibility phenomenon as a captive phenomenon observed in classical PT-symmetric structures^9^ is a special case of the ATRs. Moreover, the exceptional point occurs at *α*_*l*_ = 890, below which the UC1 bilayer was in the exact symmetry phase; otherwise, it was in the broken phase regime for both polarizations at any given angle of incidence^26^. The stability analysis of our proposed structure in Fig. 2a shows that the exceptional point is in a nonanalytical region at any angle of incidence. Because we deal with analytic functions in the upper half plane of the complex *ω*-plane, there should be no concern regarding PT symmetry-breaking transitions. For the UC2 bilayer, we showed that the PT symmetry holds solely for *α*_l_ = 2 (i.e., | *α*_g_ | = 20.86). Hence, we use these values for the loss and gain coefficients to analyze the dependency of the second-order coherence (Eq. 8) on the time delay for the quantum light transmitted through UC1 and UC2 bilayers.

**Figure 3:**
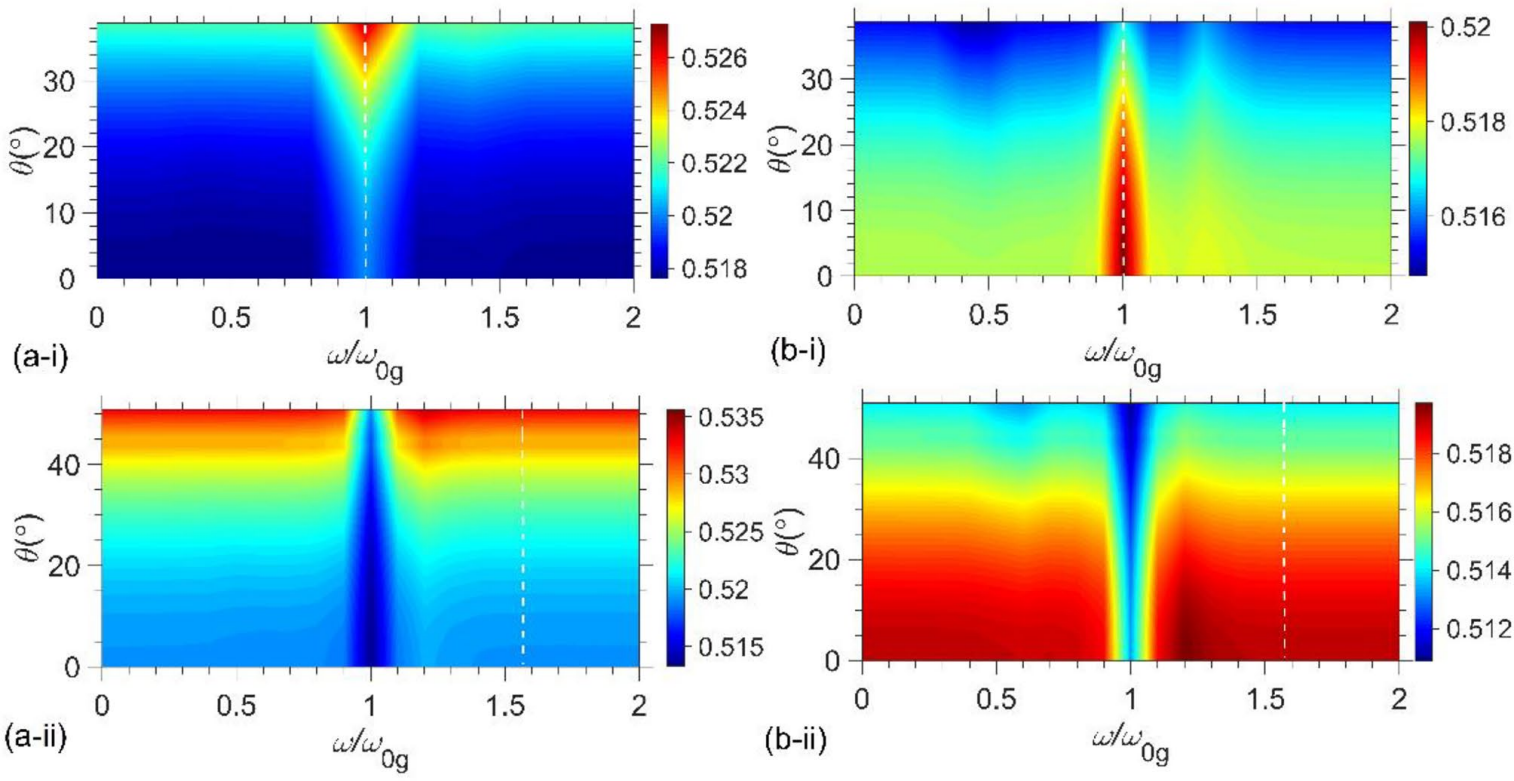
Profile of *g*_*σ*_^(2)^(0) in the ω − *θ* plane for a two-photon number state with **(a)** *s*- and **(b)** *p*-polarization transmitted through the structure made of one unit-cell designated by **(i)** UC1 at |*α*_g_ |= *α*_l_ = 24 and **(ii)** UC2 at *α*_l_ = 2 and | *α*_g_ |= 20.86. The white vertical dashed lines denote the PT-symmetric frequency *ω*_c_ / *ω*_0g_ = 1 (1.58) for UC1(UC2).

Exploiting the exact multilayer theory^35^, we depicted in Fig. 3 the distribution of the second-order correlation function for no time delay*—*i.e.*,* *g*_σ_^(2)^(0)—as functions of ω and *θ* in analytical regions for a two-photon number state transmitted through the unit-cells of (i) UC1 at |*α*_g_ | = *α*_l_ = 24 and (ii) UC2 at *α*_l_ = 2 and | *α*_g_ | = 20.86, and for (a) *s*-and (b) *p*-polarizations. Any polarization state of the electric fields of the input light, such as circular, elliptical, or linear polarizations at an arbitrary angle, can be formed by a linear combination of two orthogonal states of *s-* and *p-*polarizations by choosing the appropriate amplitude and relative phase. Indeed, all results presented here can be used for an arbitrary polarization state if both phase and amplitude parameters of two orthogonal states of *s-* and *p-*polarizations are specified. Here, the unit cells are prepared in a stable electromagnetic regime. The white vertical dashed lines denote the PT-symmetric frequency *ω*_c_ = *ω*_0g_ (1.58*ω*_0g_) for UC1(UC2).

The plots elucidate the role of the resonance and PT-symmetric frequencies in the behaviour of *g*_σ_^(2)^ (0). As can be seen from Fig. 3, always *g*_σ_^(2)^ (0) < 1, but different from that of the incident two-photon state (0.5). Therefore, the transmitted states through each unit cell partly preserve their antibunching characteristic over the entire ω−*θ* plane for both polarizations. As *θ* increases, for a given frequency, the value of *g*_σ_^(2)^ (0) for the *s*(*p*)-polarization increases (decreases) for both structures. One can attribute this variation to the differences observed in the corresponding transmission and reflection coefficients for the obliquely incident *s*-and *p*-polarized lights (see Fig. S1 in Supplement of^26^). One may use this difference in the dependencies of *g*_*σ*_^(2)^(0) for different polarization in either unit cell to control the antibunching feature of the transmitted light through non-Hermitian systems by varying the polarization. Furthermore, no significant effect can be found for the second-order coherence of exiting photons when the system is in the PT symmetry regime.

**Figure 4:**
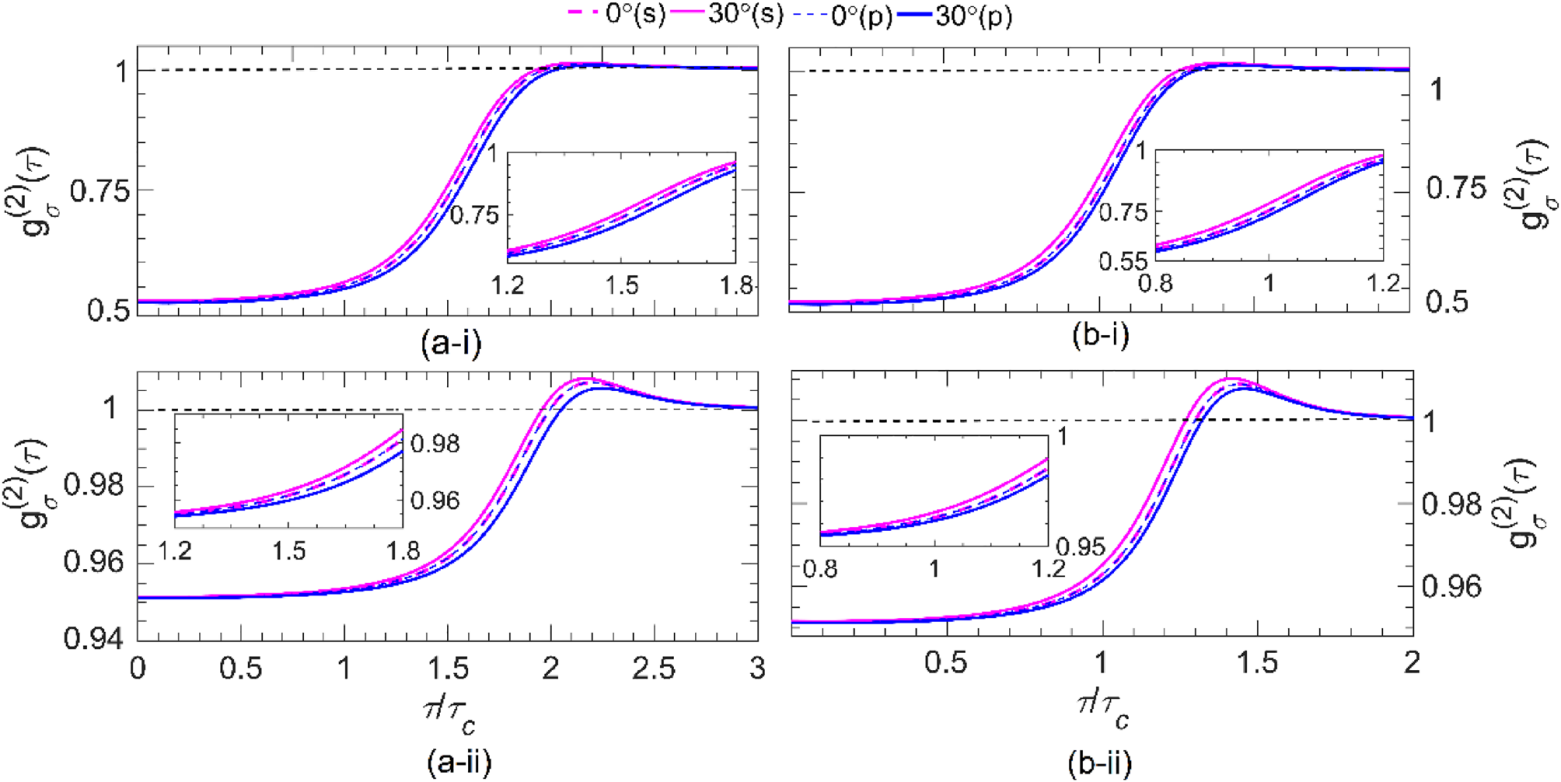
The second-order coherence *g*_*σ*_^(2)^(*τ*) versus *τ* / *τ*_c_ for **(i)** a two-photon and **(ii)** a twenty-photon wave packets with *s*-polarization (magenta) and *p*-polarization (blue) transmitted through the one unit cell of **(a)** UC1 at *α*_l_ =|*α*_g_|= 24, and **(b)** UC2 at *α*_l_ = 2 (|*α*_g_|= 20.86) for incident angles of *θ* = 0°(dashed line) and 30°(solid line). Notice that *ω*_c_ = *ω*_0g_ (1.58*ω*_0g_) for UC1(UC2). The inset in all parts shows the zoomed-in portion of the 1.2 < *τ* / *τ*_c_ < 1.8, enhancing the visibility of the ascending regime.

Figure 4 shows the plots of *g*_*σ*_^(2)^ versus *τ* / *τ*_c_ for *s*- and *p*-polarized Gaussian pulses with (i) two and (ii) twenty photons transmitted through one unit-cell of (a) UC1 with |*α*_g_ | = *α*_l_ = 24, and of (b) UC2 with *α*_l_ = 2 and | *α*_g_ | = 20.86 for incident angles of *θ* = 0° and 30°. A comparison of Fig. 4a-i, a-ii shows that the signal field transmitted through the UC1 bilayer with *α*_l_ = 24 retains its antibunching characteristic to some extent—i.e., *g*_*σ*_^(2)^(0) < *g*_*σ*_^(2)^(τ)—especially for the state with small photon numbers. For all cases considered in these two figures, *g*_*σ*_^(2)^(τ < τ*′*) is a sharply increasing function of the time delay, while τ*′* decreases (increases) with an increase in the angle of the incident for the *s*(*p*)-polarization. As *τ* increases beyond τ*′,* for any given angle, *g*_*σ*_^(2)^ increases first until it reaches a maximum value and then properly decreases to the limiting value of *g*_*σ*_^(2)^ ~ 1 at *τ* = 2*τ*_c_ (~ 2.5*τ*_c_) for the pulse with two- (twenty-) photon number states. This is because, for time delays that exceed the pulse duration *L*/*c*, a vacuum state with unity second-order coherence is detected, which is uncorrelated. The details of this phenomenon can be found in Supplementary S.1.

A comparison of Fig. 4b-i, b-ii shows a similar dependency for *g*_*σ*_^(2)^ on *τ* to those of Fig. 4a for the signal field passing through the UC2 bilayer at *α*_l_ =2. The results show that up to a particular time delay (τ*′*′) *g*_*σ*_^(2)^(τ) < 1 for any given photon number in the state. However, for all cases considered in these two figures, *g*_*σ*_^(2)^(0) < *g*_*σ*_^(2)^(τ). Thus, the antibunched feature of the transmitted state through the bilayer UC2, where the PT symmetry condition holds at | *α*_g_ | = 20.86, remains preserved for time delays of the order of τ = *τ*_c_, regardless of the photon numbers. This correlation time is nearly identical for the transmitted state through both bilayer UC1 and UC2, which can be attributed to the similar loss and gain experiences in PT symmetry conditions. However, there is still a slight difference caused by the different frequencies of the input state hitting the bilayers UC1 and UC2. This can be understood from the approximate relationship obtained for the *J*_1σ_-wave packet contribution (see Supplementary S.1). Moreover, we can observe that *g*_*σ*_^(2)^(0) tends to unity for large photon numbers due to the randomness of the outgoing photon flux. Furthermore, our observations reveal that the second-order coherence of the transmitted state through the bilayer UC1 and UC2 exhibits a slight deviation from the input state at the ATR point (| *α*_g_ | = *α*_l_ = 24). This phenomenon is a clear manifestation of the broken PT-symmetry in the quantum domain, which is in agreement with the findings of previous studies^25,35^. It is noteworthy that the results obtained in this study contradict the classical observations reported by^9^, which established that the given bilayer becomes unidirectionally invisible in the presence of obliquely incident N-photon number state of light. However, due to the presence of gain-induced quantum noise, which breaks the PT symmetry of the system regardless of the symmetry provided by the refractive index, the bilayer is no longer unidirectionally invisible. Therefore, we suggest that the implementation of PT-symmetric optical systems in the quantum domain can be achieved by switching to passive PT-symmetric systems^41^. Our findings underscore the importance of considering the impact of quantum noise on the design and operation of PT-symmetric systems in the quantum domain.

**Figure 5:**
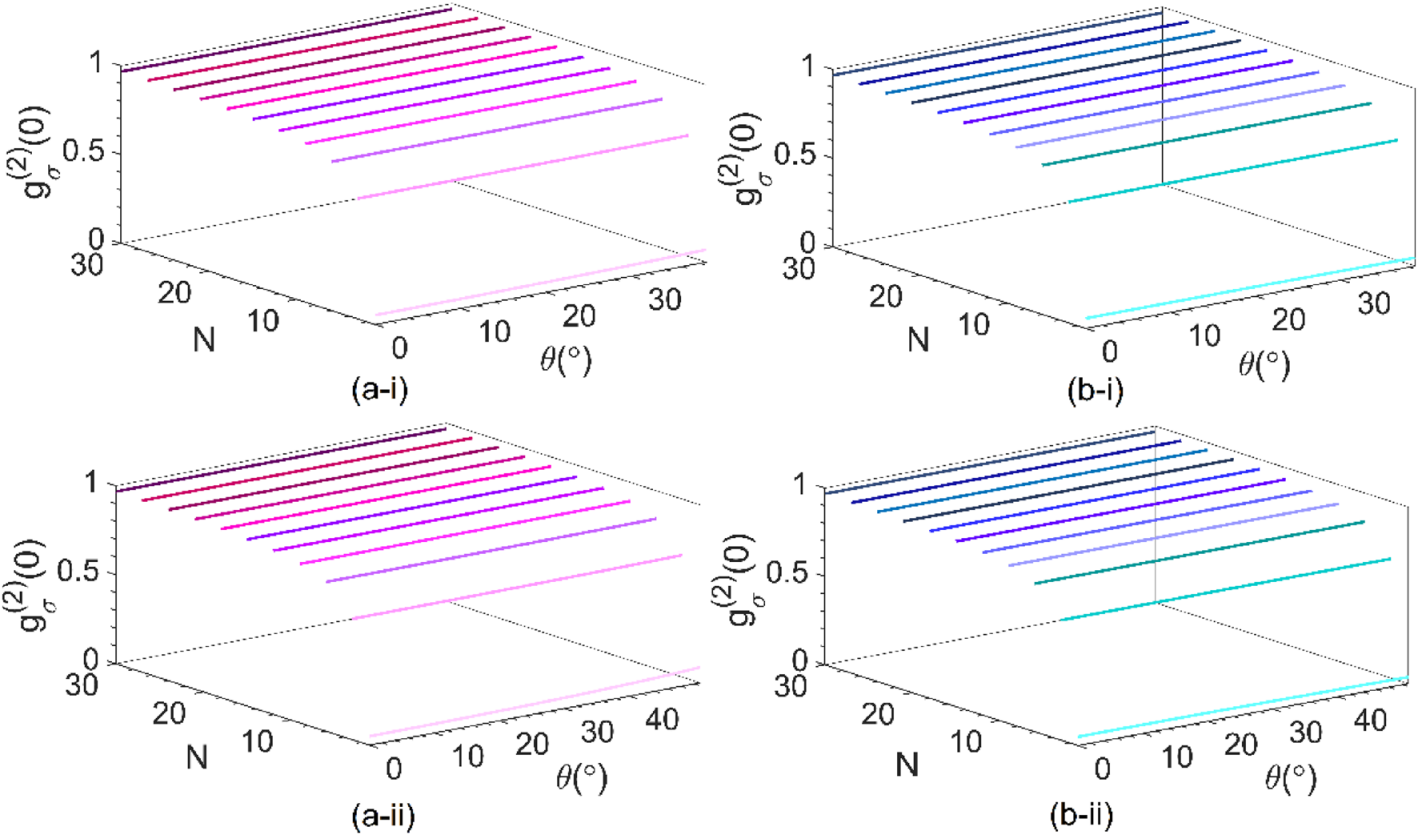
The second-order coherence *g*_*σ*_^(2)^(0) versus photon number, *N*, and incident angle, *θ,* in the analytic region*,* for incident *N*-photon-number state with *s*-polarization **(a)** and *p*-polarization **(b)** transmitted through the one unit cell of **(i)** UC1 at *α*_l_ =|*α*_g_|= 24 and 0 ≤ *θ* ≤ *39°,* and **(ii)** UC2 at *α*_l_ = 2 (| *α*_g_ |= 20.86) and 0 ≤ *θ* ≤ 51*°*. Notice that *ω*_c_ = *ω*_0g_ (1.58*ω*_0g_) for UC1(UC2).

A common feature observed in all parts of Fig. 4 is the coincidence of the magenta and blue dashed lines (representing *θ* = 0 for *s-* and *p*-polarizations), as expected for the normal incident. On the contrary, for the oblique incidence with a larger incident angle (θ > 0), lower (higher) correlations are observed for the s(p)-polarization. The observed behavior can confidently be attributed to the distinct gain-induced quantum noise fluxes for s- and p-polarizations, as explicitly explained in Supplementary S.1. As the photon number in the pulse increases from 2 to 20, g_σ_^(2)^(0) approaches unity more closely. This result is confirmed in Fig. 5, where the g_σ_^(2)^(0) is plotted versus the photon number (*N* = 1, 3, 6, …, 30) and the incident angle *θ* for (i) UC1 at *α*_l_ = |*α*_g_| = 24 and 0 ≤ θ ≤ 39° and (ii) UC2 at *α*_l_ = 2 (|*α*_g_| = 20.86) and 0 ≤ *θ* ≤ 51° for (a) *s*- and (b) *p*-polarizations. We find that by increasing the photon numbers *N*, the coherence value *g*_*σ*_^(2)^(0) increases and tends to unity independent of the angle of incidence and the polarized state of light.

**Figure 6:**
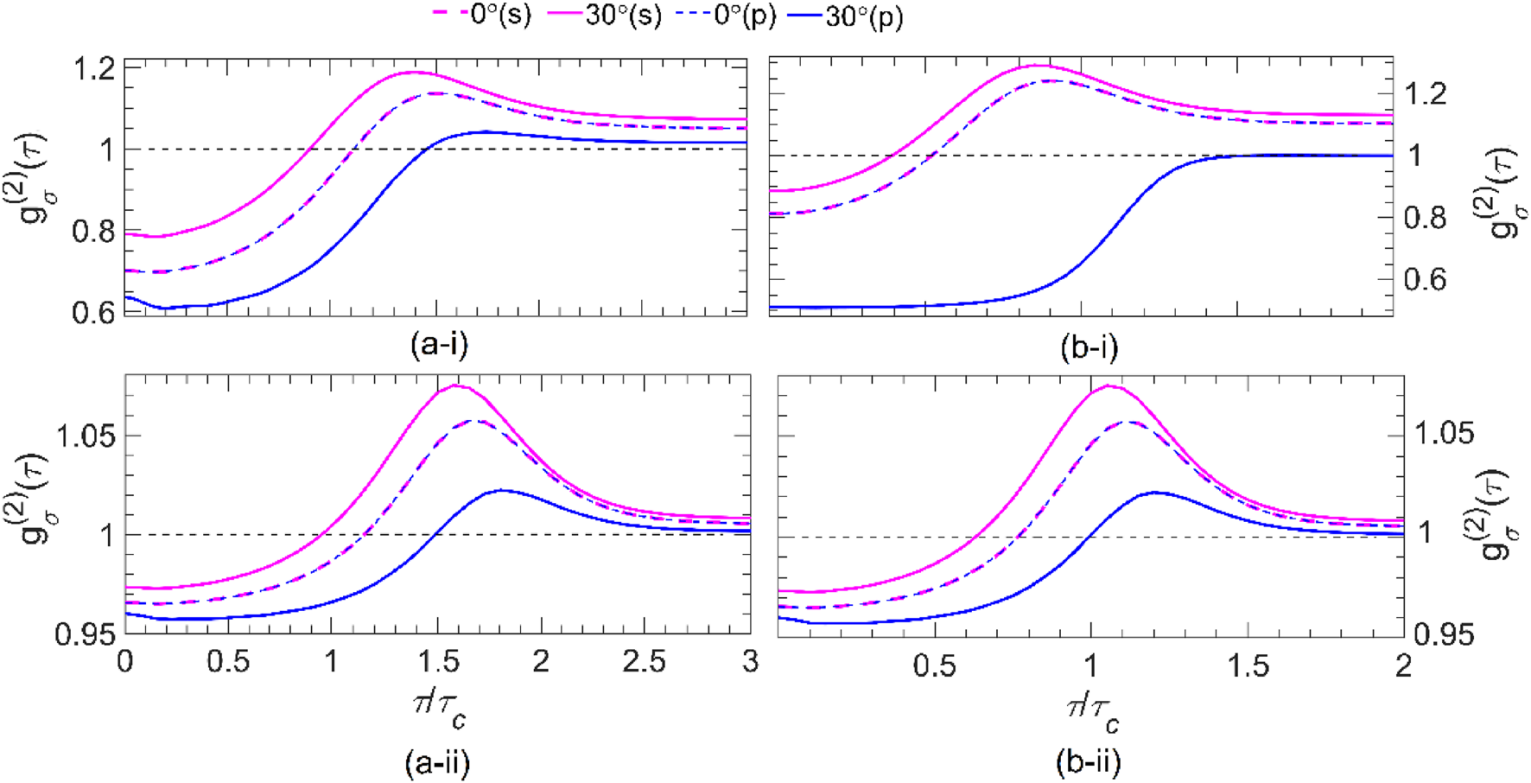
The second-order coherence *g*_*σ*_^(2) ^(*τ*) versus *τ* / *τ*_c_ for **(i)** a two-photon and **(ii)** a twenty-photon wave packets with *s*-polarization (magenta) and *p*-polarization (blue) transmitted through the four unit-cell designated by **(a)** UC1 at *α*_l_ =|*α*_g_|= 24 and **(b)** UC2 at *α*_l_ = 2 (| *α*_g_ |= 20.86) for incident angles of *θ* = 0°(dashed line) and 30°(solid line). Notice that *ω*_c_ = *ω*_0g_ (1.58*ω*_0g_) for UC1(UC2).

To further investigate the effects of quantum noises originating from the gain layers, here, we consider both non-Hermitian structures of Fig. 1 (UC1 and UC2), each consisting of four unit cells, and calculate the dependency of the second-order coherence (9) on the time delay (*τ* / *τ*_c_) as plotted in Fig. 6 for (i) two- and (ii) twenty-photon Gaussian pulses with *s*- and *p*-polarizations propagating through (a) UC1 at |*α*_g_ | = *α*_l_ = 24, and (b) UC2 at *α*_l_ = 2 and |*α*_g_ | = 20.86. Again, we focused on two selected angles, *θ* = 0 and 30°. The results of Fig. 6a-i and a-ii show that the transmitted light is antibunched at *α*_l_ = 24 depending on the polarization and the angle of incidence, regardless of the photon number. However, the second-order coherence of the transmitted light for no time delay *g*_σ_^(2)^(0) gets closer to unity for two- and twenty-photon Gaussian pulses. Moreover, as the results in these two figures show, the second-order coherence spectrum for *s*(*p*)-polarizations at *θ* =30° is larger (smaller) than that of the normal incident (i.e., for any given *τ* / *τ*_c_, *g*_σ_^(2)^(30°) > *g*_σ_^(2)^(0°) for s-polarization and *g*_σ_^(2)^(30°) < *g*_σ_^(2)^(0°) for p-polarization). Furthermore, as *τ* / *τ*_c_ increases*,* *g*_σ_^(2)^ increases sharply up to a maximum value around *τ* ~ 1.5*τ*_c_, followed by a decrease, approaching the limiting value of near unity for τ/*τ*_c_ >1.5. Similar to Fig. 4 and Fig. S3, the magenta and blue dashes coincide over the given range of *τ*, indicating that the quantum state normally incident upon a planar structure is polarization-independent. Fig. 6b-i and b-ii depict similar *g*_σ_^(2)^(τ) plots for the given UC2 structure under the same conditions as for Fig. 6a-i and a-ii. These results show that the transmitted state through the UC2 structure retains its antibunching characteristics less, regardless of the photon number. However, we can observe that for large photon numbers, *g*_*σ*_^(2)^ (0) approaches closer to unity than for UC1. A careful comparison of each plot in Figure 6 with its counterpart (i.e., with the same *α*_l_) reveals that as the number of constituent unit cells in either structure increases, the corresponding *g*_*σ*_^(2)^(0) increases, independent of polarizations, degrading the quantum antibunching feature of the transmitted state drastically due to the significant increase in the quantum noises within the structure originated from the gain layers at 0 K.

## Conclusion

We have considered two sets of dispersive non-Hermitian multilayered structures: UC1 with identical background materials constituting the gain and loss layers in the unit cell; and UC2 with unidentical background materials for the loss and gain layers. So, the PT-symmetric necessary condition for UC1 (UC2), with *m* unit cells composed of gain/loss layers with identical (unidentical) background permittivities, is *ω* = *ω*_PT_ = *ω*_0g_ (1.58 *ω*_0g_). Illuminating both structures obliquely using the *s*- and *p*-polarized quantum states of light, we have investigated the second-order coherence of the transmitted quantum lights through each. We used the Lorentz model to incorporate the dispersion and dissipation/amplification effects of the non-Hermitian structure to evaluate the second-order coherence of the transmitted light. We have observed that the PT-symmetric condition for UC1 holds for a wide range of loss/gain strengths*,* whereas PT-symmetry for UC2 was established only for a specific loss coefficient. In other words, the experimental realization of the UC1 seems more practical for PT symmetry. The results for *m* = 1 show that the nonclassical antibunching properties of the incident quantum states of light are partly preserved over a range of the *α*_*l*_−*θ* plane after transmitting through both sets, especially when *N*-photon number state and quantum vacuum state are incident from the left or right side of the structures in the stable regime. In the quantum domain, the unidirectional invisibility character of PT-symmetric structures cannot be realized when probed by the *N*-photon number state of light. This conflicts with the classical results of^9^. Furthermore, we have shown that for large photon numbers in the input state or a large number of unit cells constituting the multilayered structure, the antibunching feature of the transmitted state degrades for either polarization. From this perspective, the realization of PT symmetry in quantum optics is very fragile regarding the antibunching feature of outgoing photons. In general, PT symmetry cannot be implemented in quantum optics because of the deep dependence on the nature of the quantized light, the polarization of the incident light at oblique incidence, the number of unit cells and type of structure, and how the photon state is prepared. Our research holds the potential to revolutionize the design of linear optical networks for quantum information processing and advanced sensing applications. By exploiting non-Hermiticity, we aim to inspire a new approach to the design of such networks. Furthermore, our work on PT-symmetric quantum optics offers a promising avenue for investigating plasmonic structures where losses can be compensated by gain. This research is crucial in pushing the boundaries of quantum information processing and sensing, and we are confident that our findings will pave the way for exciting new developments in the field.

### Supplementary Information

Supplementary Information.

## Data Availability

Data underlying the results presented in this paper are not publicly available at this time but may be obtained from the corresponding author upon reasonable request.
