# Supplementary material for: The second-order coherence analysis of number state propagation through dispersive non-Hermitian multilayered structures
Source: Sci Rep. 2024 Mar 5;14:5399. doi: 10.1038/s41598-024-55777-0 (PMC10914838; doi:10.1038/s41598-024-55777-0)
Supplement: Supplementary file 1 — Supplementary Information. [file 41598_2024_55777_MOESM1_ESM.docx]

**Supplementary:**

The Second-Order Coherence Analysis of Number State Propagation through Dispersive Non-Hermitian Multilayered Structures

**Elnaz Pilehvar^1^, Ehsan Amooghorban^2,3^, and Mohammad Kazem Moravvej- Farshi^1,*^**

^1^ Nano Plasmo-Photonic Research Group, Faculty of Electrical and Computer Engineering, Tarbiat Modares University, P.O. Box 14115-194, Tehran 1411713116, Iran.

^2^ Faculty of Science, Department of Physics, Shahrekord University, P.O. Box 115, Shahrekord 88186-34141, Iran.

^3^ Nanotechnology Research Group, Shahrekord University, Shahrekord, Iran.

^*^ Email: [moravvej@modares.ac.ir](mailto:moravvej@modares.ac.ir)

Section S.1 in this supplementary presents the details of Eq. (9) given in the manuscript, and Section S,2 describes the second-order coherence of different two-photon states, supporting the manuscript content**.**

**S.1. Details of Eq. (9)**

The contribution of the transmitted state is described by the integral

 (S1)

and that of the noise flux by

 (S2)

where *q* is the generic time parameter. The explicit form of the noise expectation value$\left\langle\hat{F}_{R\sigma}^{\dagger}(\omega,k)\hat{F}_{R\sigma}(\omega,k)\right\rangle$ is given in Eq. (A19) of [35]. To further support our argument, we would like to introduce a simplified form for the *J*_1σ_-wave packet contribution. By approximating the term $\omega^{1/2}\text{ }T_{\sigma}(\omega,\text{k})$ in the integrand of the integral (S1), we can evaluate it and obtain $J_{1\sigma}\left( q,\theta\right)\simeq\sqrt{{\hbar\omega_{c}}/{2\pi\varepsilon_{0}LA}}\text{ }{(2\pi)}^{-1/4}\text{ }T_{\sigma}(\omega_{c},\text{k})\exp[-i\omega_{c}q-c^{2}q^{2}/L^{2}]$ that clearly demonstrates the vanishing of the wave packet contribution in the second-order correlation function (9) at a large time delay. However, this term plays an important role in the evaluation of the function for the limiting case of zero-time delay in evaluating$g_{\sigma}^{(2)}$.

Equation (9) in conjunction with Eqs. (S1) and (S2) reveal the distinct dynamics displayed in Figs. 4, 5, and 6 for UC1 and UC2 are contingent on the interplay between the wave packet contribution via the *J*_1σ_ term, and the quantum noise contribution through the *J*_2σ_ term. In physical terms, unlike the loss layers that are devoid of any noise flux at zero temperature, the noise flux contribution stems from the gain layers owing to the perfect population inversion of the atoms.

Figure S1 (S2) displays the transmittance *T_𝜎_* = | t*_𝜎_*|^2^, right reflectance R_R_*_𝜎_* = |r_R_*_𝜎_*|^2^, and noise flux $\left\langle\hat{F}_{R\sigma\text{ }}^{\dagger}\text{ }\hat{F}_{R\sigma\text{ }} \right\rangle$ as a function of the incident angle, *θ,* for s-polarized (magenta) and p-polarized (blue) light. The light impinges upon one (four) unit-cell slab designated by (a) UC1 at |*α*_g_| =*α*_l_ = 24 and (b) UC2 at *α*_l_ = 2 and | *α*_g_ | = 20.86. It is important to note that for UC1(UC2), *ω* = *ω*_0g_ (1.58*ω*_0g_). The results displayed in Figs. S1 (a-i) and (b-i) reveal that for any given value of α_l_, and small to moderate angle of incidence, the right reflectance and transmittance for the one unit-cell slabs of UC1 and UC2 for both polarizations approach R_R_ ≈ 0 and T ≈ 1. However, the behavior of parameters *R*_R_ and *T,* shown in Figs. S2 (a-i) and (b-i) slightly differ for *s*- and *p*-polarized light even at small incident angles in the four-unit cell slabs of UC1 and UC2. Moreover, a comparison of results, shown in the panels marked by (ii) in Figures S1 and S2, reveals that the noise fluxes generated in both single and four unit-cell slabs of UC2 are considerably small over the entire range of incident angles. This is because the center frequency of the Gaussian wave packet, which is the frequency at which the PT-symmetry condition is fulfilled, is far from the resonance frequency of the loss and gain layers constituting the UC2 slabs. On the other hand, the coincidence of frequencies of PT-symmetry and incident wave with the resonant frequency of the gain and loss layers constituting the UC1 slabs makes the noise flux generated in UC1 an increasing function of incident angle. Nonetheless, although the noise flux is relatively small at small angles, it is still higher than that of UC2. Hence, for UC1 and UC2 bilayers, when *θ <*0°*,* —i.e., falling within the stable regime, — the wave packet’s contribution dominates over the noise contribution for both polarizations (as per Equation (9)). Therefore, when we consider the limiting case of zero time-delays, Eq. (9) reduces to $g_{\sigma}^{(2)}\left( t_{r}=0,\tau=0,\theta\right)\approx\left( N-1 \right)/{N<1}$, regardless of the polarization and angle of the incident. Thus, the transmitted light through UC1 and UC2 bilayers would be antibunched for both polarizations. However, the second-order coherence of the output mode is slightly different from its initial value before hitting the bilayer structures. A comparison of different *N*-photon number states shows that for *N* = 2, the $g_{\sigma}^{(2)}\left( t_{r}=0,\tau=0,\theta\right)\to0.5$, as expected from the results depicted in Figs. 3 and 4(a), while for *N*= 20, $g_{\sigma}^{(2)}\left( t_{r}=0,\tau=0,\theta\right)$→0.95 (as per Fig. 4(b)). These results are consistent with the results shown in Fig. 5, where $g_{\sigma}^{(2)}\left( t_{r}=0,\tau=0,\theta\right)$ is independent of polarization and incident angle.

The significant noise contributions for the UC1 and UC2 slabs that consist of four unit cells (observed in Figs. S 2(a-ii) and S2(b-ii), is due to the considerable number of gain layers in both slabs, simplifying Eq. (9) to $g_{\sigma}^{(2)}\approx2-{N\left( N+1 \right)\left| J_{1\sigma}\left( t_{r},\theta\right) \right|^{4}}/{\left[ N\left| J_{1\sigma}\left( t_{r},\theta\right) \right|^{2}+J_{2\sigma}\left( 0,\theta\right) \right]^{2}}$for zero time delays, which is slightly greater than ${(N-1)}/N$ approximated for the bilayer structures. Moreover, the noise flux generated for s-polarization is more than that for p-polarization (see panels (ii) in Figs. S1 and S2). The differences in noise fluxes between s- and p-polarizations can be readily inferred from the behaviors of the transmittance *T* and right reflectance *R_R_* of the bilayers UC1 and UC2, which are in turn related to the effective noise flux. In this effective description, sub-wavelength structures in the quantum domain can be characterized by a single homogeneous effective slab with two effective parameters of refractive index and noise photon distribution. For a more detailed understanding of the quantum optical effective-medium theory, we refer the interested readers to Refs. [35,36]. As shown in Figs. 3, 4, and 6, the $g_{\sigma}^{(2)}$ is larger for s-polarization than for p-polarization. Consequently, as the number of bilayers forming the slab increases, the noise contribution, especially for the s-polarization, hinders the quantum correlation of the transmitted state and diminishes the antibunching character.

**
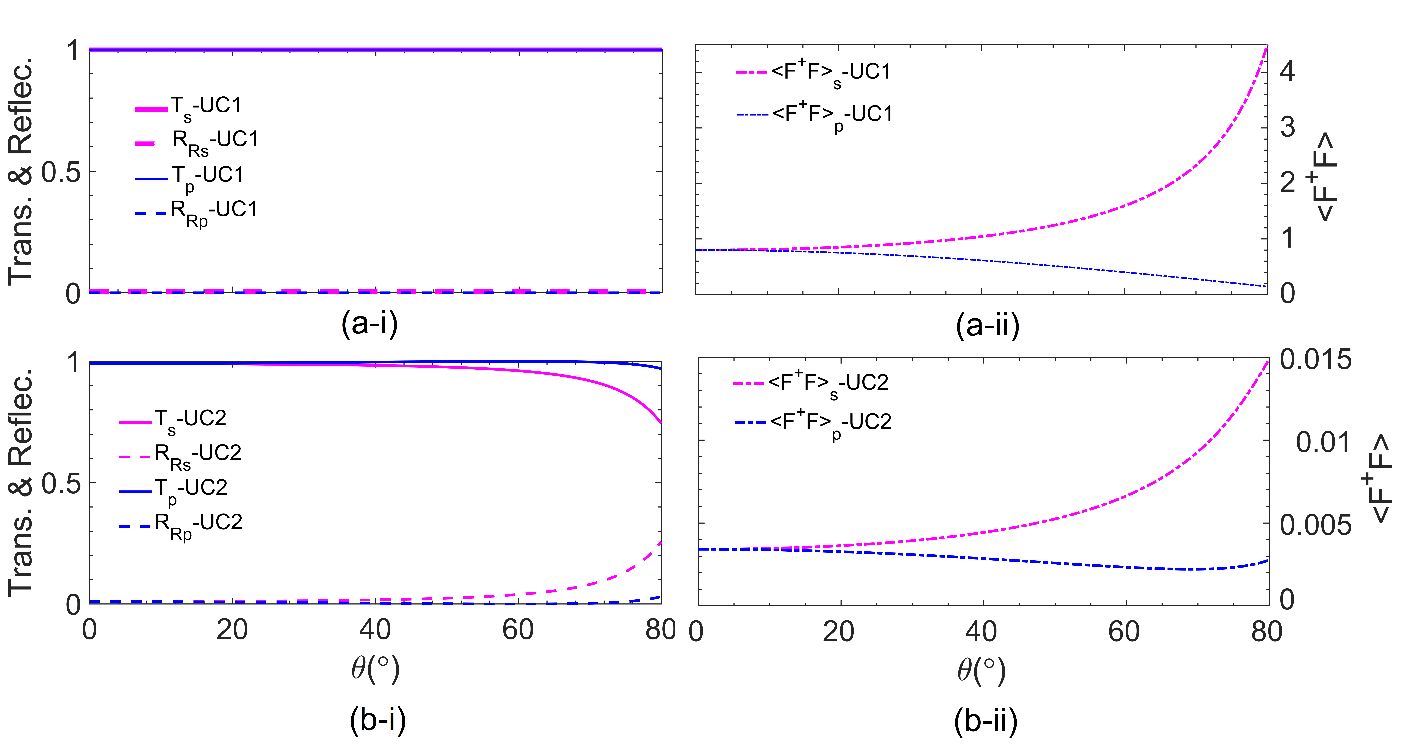
**

**Figure S1.** **Figure S1.** The **(i)** transmittance *T* (solid line), right reflectance *R*_R_ (dashes), **(ii)** noise flux $\left\langle\hat{F}_{R\sigma\text{ }}^{\dagger}\text{ }\hat{F}_{R\sigma\text{ }} \right\rangle$ (dots-dashes), vs. the incident angle *θ* for *s-*polarized (magenta) and *p-*polarized (blue) light impinging upon a one unit-cell of **(a)** UC1 at |*α*_g_| =*α*_l_ = 24 and **(b)** UC2 at *α*_l_ = 2 and | *α*_g_ | = 20.86


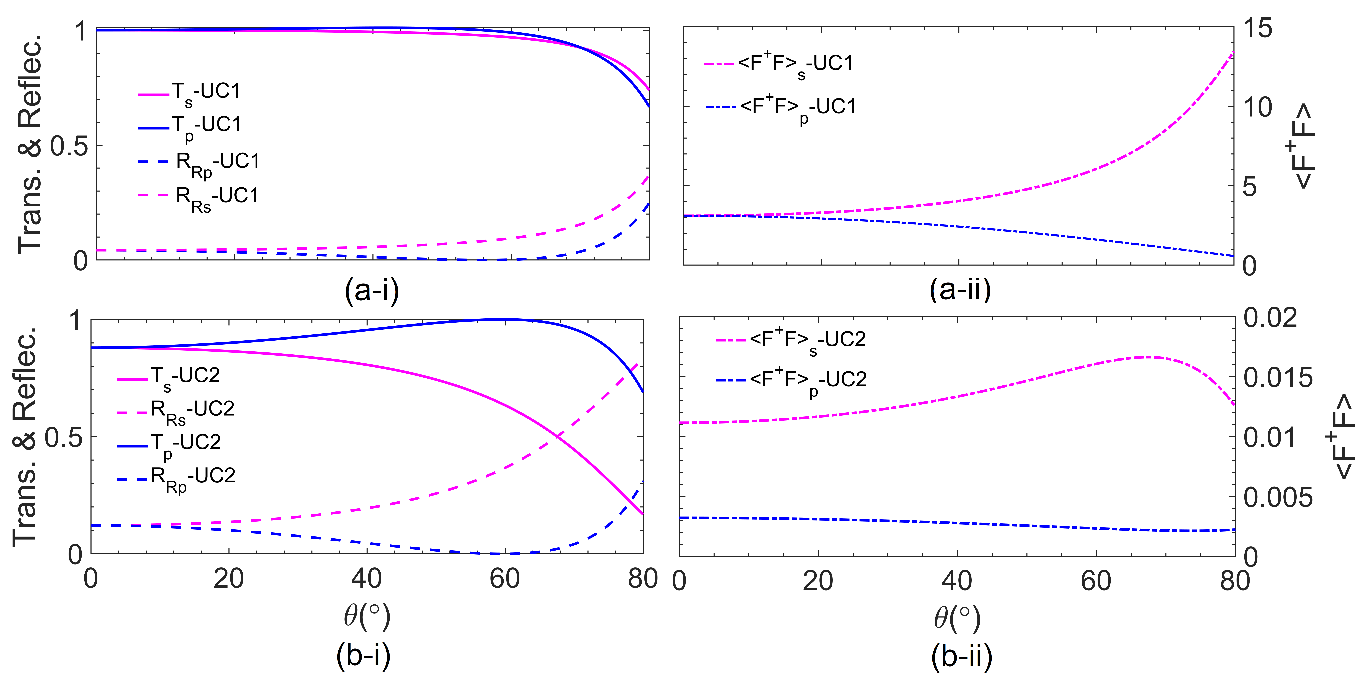


**Figure S2.** The **(i)** transmittance *T* (solid line), right reflectance *R*_R_ (dashes), **(ii)** noise flux $\left\langle\hat{F}_{R\sigma\text{ }}^{\dagger}\text{ }\hat{F}_{R\sigma\text{ }} \right\rangle$ (dots-dashes), vs. the incident angle *θ* for *s-*polarized (magenta) and *p-*polarized (blue) light impinging upon a four unit-cell of **(a)** UC1 at |*α*_g_| =*α*_l_ = 24 and **(b)** UC2 at *α*_l_ = 2 and | *α*_g_ | = 20.86

For large time delays *τ* ≫ *τ*_c_, the wave packet contribution $\left|J_{1\sigma}\left(t_{r},\tau\gg\tau_{c},\theta\right)\right|$ is negligible, and the degree of second-order coherence (9) is reduced to $g_{\sigma}^{(2)}\approx1+{\left| J_{2\sigma}\left( \tau,\theta\right) \right|^{2}}/{J_{2\sigma}\left( 0,\theta\right)\left[ N\left| J_{1\sigma}\left( t_{r},\theta\right) \right|^{2}+J_{2\sigma}\left( 0,\theta\right) \right]}.$ Given that for *τ* ≫ *τ*_c_, the noise contribution satify the relation $\left| J_{2\sigma}\left( \tau,\theta\right) \right|\ll J_{2\sigma}\left( 0,\theta\right)$, the second term of the resulting equation becomes zero, and $g_{\sigma}^{(2)}(\tau\gg\tau_{c})\to1$. This result is expected because, for time delays larger than the pulse duration *L/c*, which is equal to the mean coherence time (*τ*_c_), an uncorrelated vacuum state with unity second-order coherence is detected.

**S.2 The second-order coherence of different two-photon states**

Figure S3 shows the plots of *g_𝜎_*^(2)^(0) versus 𝛼*_l_* for two-photon Gaussian pulses (i) | 2 ⟩| 0 ⟩, (ii) | 0 ⟩| 2 ⟩, and (iii) (| 2 ⟩| 0 ⟩ +| 0 ⟩| 2 ⟩ ) / √2 transmitted through one unit-cell designated by (a) UC1 at |*α*_g_| =*α*_l_ = 24, and (b) UC2 at *α*_l_ = 2 and | *α*_g_ | = 20.86 at the selected angles θ = 0°, 30°, and 60° for both polarizations. We restrict ourselves to the *α*_l_ regime where *g*^(2)^(0) for all selected angles is in the analytic region — i.e., *α_l_ <* 18.5. One can create entangled states through various methods that depend on the quantum system and the desired state. In our recent work, we followed the approach detailed in [40] to generate the entangled state $(|0\rangle|2\rangle+|2\rangle|0\rangle)/\surd2$. We utilized the input state $|1\rangle|1\rangle$ to transmit through a symmetric and lossless 50:50 beam splitter. The interference effects of this process caused the input number state to transform into the desired entangled state. Then, we used this entangled state as one of the input modes for the structure illustrated in Fig. 1.

As can be observed in Figures S3(a-i) and S3 (b-i), the signal field transmitted through both sets is antibunched for the given values of *α_l_* and θ, for either polarization — i.e., *g_𝜎_*^(2)^(0) < 1. A comparison of the plots S3(a-ii) and S3(a-iii) shows that there is an *α_l_* range over which the output light is bunched (i.e., *α_l_*′ < *α_l_* < *α_l_*′′), while it is antibunched in the remaining range of *α_l_* values (i.e., *α_l_* < *α_l_*′ and *α_l_* > *α_l_*′′), wherein *g*_𝜎_^(2)^(0) ≤ 1. The values of loss coefficients *α_l_*′ and *α_l_*′′ corresponding to different *θ* values for both polarizations are tabulated in Table S1. On the contrary, Figures S3(b-ii) and S3(b-iii) show that over the entire ranges of the given *α_l_* and for both polarizations, the transmitted states through UC2 do not retain their initial antibunching property.

A comparison of the plots in Figures S3(a) and S3(b) reveals that the UC1 bilayer can retain the antibunching characteristics of the incident state better than UC2, especially when the restructures are illuminated by the interchanged state | 0 ⟩| 2 ⟩, or the entangled state (| *2* ⟩| 0 ⟩+| 0 ⟩| 2 ⟩)/$\surd2$. Moreover, the propagation of normal incident quantum states through two sets of the structure is polarization-independent, as expected. Nonetheless, the deviation of magenta dotted or solid lines from their blue counterparts in each figure (i.e., *θ*> 0) indicates that as the *θ* increases, *g*^(2)^(0) for the *s*(*p*)-polarization compresses (expands) towards smaller (larger) *α*_l_.

Bearing these in mind, it seems that the incident state | 2 ⟩| 0 ⟩ is a more suitable state for probing the antibunching characteristic of the outgoing photons from the proposed non-Hermitian structures. Since, the transmitted state through both sets remains antibunched for the given values of *α_l_* and θ, for either polarization.

**Table S1.** The values of loss coefficients (*α_l_* and *α_l_*′′ ) for which of the output from the one unit-cell of UC1 transits between bunched and antibunched, for the two-photon Gaussian pulses from the right side of the structure | *0* ⟩| *2* ⟩ and entangled pulse (| *2* ⟩| *0* ⟩ +| *0* ⟩| *2* ⟩ ) / √2 at selected θ = 0°, 30°, and 60°given in Figure S3.

| *Θ°*(𝝈) | 0° | 30°(s) | 60°(s) | 30°(p) | 60°(p) |
| --- | --- | --- | --- | --- | --- |
| *α_l_*′ (Figure 4(a-i)) | 0.2 | 0.2 | 0.2 | 0.2 | 0.2 |
| *α_l_*′′ (Figure 4(a-i)) | 6.5 | 6 | 4 | 7.5 | 13 |
| *α_l_*′ (Figure 4(b-i)) | 0.14 | 0.14 | 0.14 | 0.14 | 0.14 |
| *α_l_*′′ (Figure 4(b-i)) | 9.1 | 7.2 | 4.8 | 9 | 16.8 |


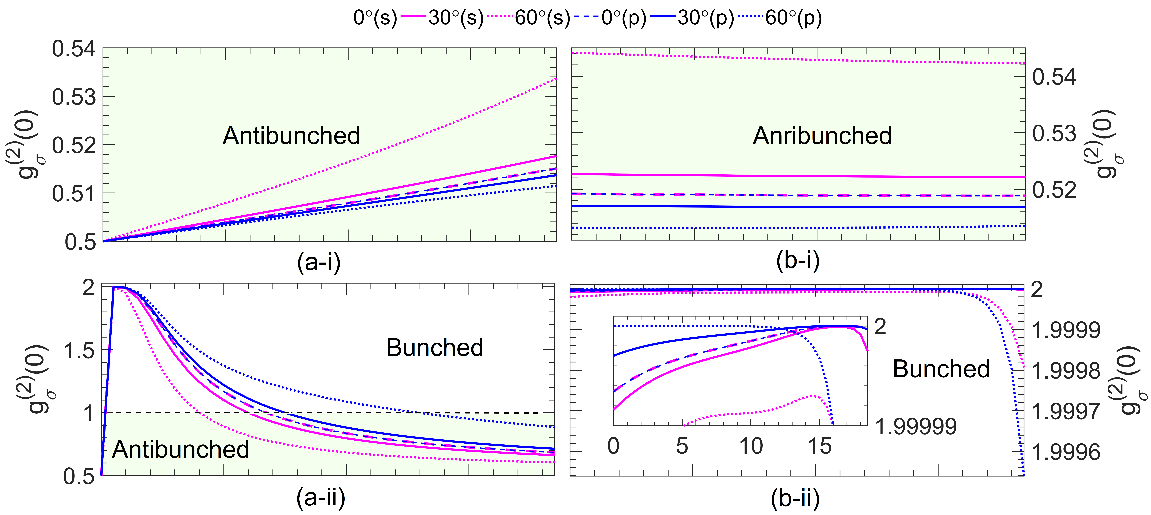


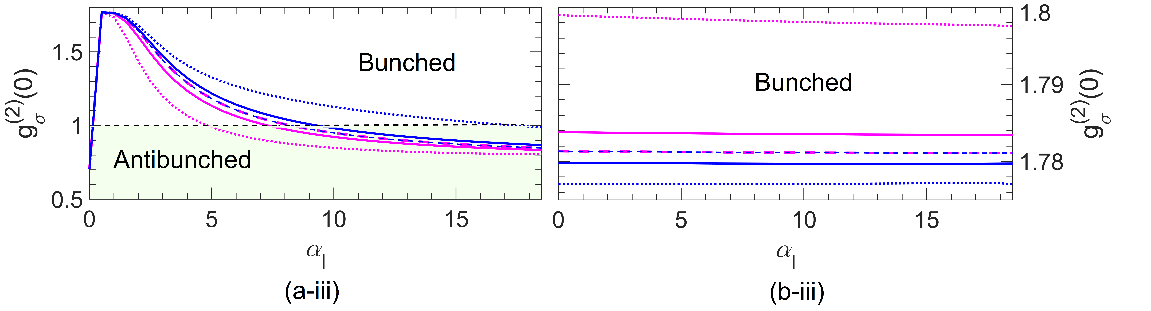


**Figure S3.** The second-order coherence *g_𝜎_*^(2)^(0) versus *α*_l_ for a two-photon wave packet **(i)** | 2, 0 ⟩, **(ii)** | 0, 2 ⟩, and **(iii)** (| 0, 2 ⟩+| 2, 0 ⟩)/√2 with *s*-polarization (magenta) and *p*-polarization (blue) transmitted through the one unit-cell of **(a)** UC1 at *ω*_c_ = *ω*_0g_ and **(b)** UC2 at *ω*_c_ = 1.58*ω*_0g_, for incident angles of θ = 0°(dashed line), 30°(solid line), and 60°(dotted line) where all plots are in the analytic region (*α_l_ <* 18.5). The region shaded light green in part (a) represents the antibunched regime.
